# Surface Wave Enhanced Sensing in the Terahertz Spectral Range: Modalities, Materials, and Perspectives

**DOI:** 10.3390/s19245505

**Published:** 2019-12-13

**Authors:** Mathieu Poulin, Steven Giannacopoulos, Maksim Skorobogatiy

**Affiliations:** Department of Engineering Physics, Ecole Polytechnique de MontréalC.P. 6079, Succ. Centre-Ville, Montreal, QC H3C3A7, Canada; mathieu-2.poulin@polymtl.ca (M.P.); steven.giannacopoulos@polymtl.ca (S.G.)

**Keywords:** terahertz band, surface waves, amplitude sensing modality, phase sensing modality, surface plasmon polaritons, surface phonon polaritons, Zenneck waves, spoof plasmons

## Abstract

The terahertz spectral range (frequencies of 0.1–10 THz) has recently emerged as the next frontier in non-destructive imaging and sensing. Here, we review amplitude-based and phase-based sensing modalities in the context of the surface wave enhanced sensing in the terahertz frequency band. A variety of surface waves are considered including surface plasmon polaritons on metals, semiconductors, and zero gap materials, surface phonon polaritons on polaritonic materials, Zenneck waves on high-k dielectrics, as well as spoof surface plasmons and spoof Zenneck waves on structured interfaces. Special attention is paid to the trade-off between surface wave localization and sensor sensitivity. Furthermore, a detailed theoretical analysis of the surface wave optical properties as well as the sensitivity of sensors based on such waves is supplemented with many examples related to naturally occurring and artificial materials. We believe our review can be of interest to scientists pursuing research in novel high-performance sensor designs operating at frequencies beyond the visible/IR band.

## 1. Introduction

The extreme spatial confinement of surface plasmon polaritons (SPPs) in the visible spectral range has led to a revolution in sensing [1]. Plasmonic waves propagate along the metal/dielectric interface, featuring a deeply sub-wavelength (100 nm–1 µm) modal size [2,3,4], and optical properties that are highly sensitive to changes in the refractive index or geometrical structure of the analyte in the direct vicinity of the metal surface. These properties make surface plasmon polaritons ideal for high-sensitivity surface sensing applications and characterization of deeply sub-micron-sized objects (such as proteins, cell organelles, viruses, etc.) that have been immobilized or captured at the metal surface [5]. At the same time, due to extreme confinement of plasmons in the visible spectral range, they are not optimal for measuring bulk properties of a neighbouring analyte nor characterization of the larger super-1 µm size objects (such as bacteria, dust particles, etc.).

Recently, the THz spectral range (frequencies of 0.1–10 THz, wavelengths of 3 mm–30 µm) has emerged as the next frontier in non-destructive imaging and sensing. Key advantages when operating in the THz band are the ability to detect the complex electric field of THz waves (phase and amplitude), availability of both pulsed (sub-1 ps) and continuous wave (CW) THz systems, as well as ability of conducting dynamic measurements with sub-1 ms temporal resolution [6]. Additionally, relative transparency of dry dielectrics to THz radiation, strong absorption of THz waves by water and other polar solutions, as well as the non-ionizing nature of THz light enable many applications in non-destructive 2D imaging, 3D tomography, industrial sensing, bio-imaging/sensing, and security [7]. In the context of surface sensing, due to a relatively large size of THz waves, they can be used, in principle, for detection, characterization, and dynamic monitoring of changes in super-micron-sized objects (such as bacteria, cells, micro-organisms and some organelles, fine powders, aerosols, suspensions, condensates, etc.) conveniently localized on a flat surface [8]. Unfortunately, generation of highly-confined surface modes using simple metals (ex. gold, silver) proved to be challenging in the THz spectral range. This is because, in the THz band, the dielectric constant of most simple metals is almost purely imaginary in contrast to the visible spectral range where it is almost purely real and negative. While excitation of surface waves at the metal surface in THz is still possible, such waves tend to significantly extend into the neighboring dielectric (100–1000 wavelengths) [9]. Such surface modes also tend to have low loss (defined by the dielectric material) and can be used in principle for bulk sensing applications.

The main goal of this paper is to go beyond surface plasmon polaritons at the interface with simple metals. In what follows we present a critical comparative analysis of alternative materials, different surface wave types, and sensor modalities that could be used to design surface wave enhanced sensors operating in the THz spectral range. We demonstrate that very different surface sensing modalities can be realized in the THz band that are characterized by different degrees of field localization in the vicinity of the material surface. Thus, true surface sensors with sub-wavelength probing depths can be realized using surface plasmon polaritons on semiconductors and zero-gap materials, surface phonon polaritons on polaritonic materials, as well as spoof plasmons and spoof Zenneck waves on structured materials with high absolute values of the dielectric constant. At the same time, bulk sensors that utilize surface waves with super-wavelength probing depths can be realized using surface plasmon polaritons on simple metals, as well as Zenneck waves on high-k dielectrics and polaritonic materials.

The paper is structured as follows: we first present a theoretical foundation for the amplitude and phase-based sensor detection modalities, and then develop expressions for the corresponding sensor sensitivities. We then present a brief theory of surface waves, their classification, and their optical properties such as modal size and modal propagation distance. Next, for each wave type we consider natural or artificial materials that can be used for its realization in the THz spectral range, as well as surface wave optical properties as related to such practical materials. Finally, we summarize our findings by classifying different wave types according to their potential in the surface or bulk sensing applications as well as projected sensitivity.

## 2. Amplitude and Phase-Based Sensor Modalities Using Surface Waves

Here we consider a subset of sensors that use surface waves to detect and quantify changes in the refractive index of a neighboring analyte. The wave is assumed to propagate at the interface between a functional material (such as metal, semiconductor, etc.) and a simple dielectric (analyte) characterized by the refractive index $n_{d}$. The complex effective refractive index of a surface mode is defined as $n_{eff}\left(n_{d}\right)$ and both its real part (phase) and its imaginary parts (loss) are sensitive to the variation in the refractive index of a dielectric medium. 

As complex dispersion relations for many surface waves are known analytically, then surface wave sensitivity to changes in the analyte refractive index can also be derived in the analytical form. This allows intimate understanding of the effects of material and design parameters on sensor performance. Importantly, the study of surface wave sensitivity should be supplemented with the analysis of surface wave extent into the analyte region, which determines the sensor probing depth and allows its characterization (somewhat ambiguously) as the “surface” or “bulk” sensor. 

Alternatively, one can perform sensor characterization by studying surface wave sensitivity to changes in the thickness of a thin layer deposited at the interface. In fact, this modality is directly relatable to many practical implementations of the surface sensors. Theoretically, however, this arrangement is somewhat cumbersome to analyze as one must specify the refractive index the initial thickness of the deposited layer. The addition of these two free parameters makes analysis cumbersome and more graphical than analytical, which is the main reason why we chose a more theoretically elegant approach for the characterization of sensor sensitivity with respect to changes in the analyte refractive index complemented by the study of the surface wave probing depth. 

### 2.1. Amplitude Sensing Modality

**Figure 1:**
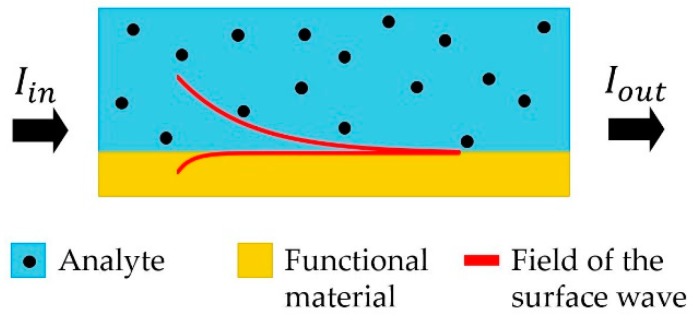
Schematic of an amplitude sensitive detection strategy. A light source is used to excite the surface mode at the interface between functional material and analyte. Variations in the transmitted amplitude is used to monitor changes in the analyte refractive index.

Amplitude sensing modality offers the simplest sensor implementation as it only requires monitoring of the sensor transmission amplitude. A simplified schematic of an amplitude-based sensor is presented in Figure 1. The amplitude sensing detection modality is mostly used to detect variations in the imaginary part of the analyte refractive index.

Within the amplitude-based modality, a small variation in the refractive index δn of the analyte can be quantified by measuring changes in the modal transmission loss $\alpha \left(n_{d}\right)$, which is, in turn, related to the imaginary part of the surface mode effective refractive index $n_{eff}^{i}\left(n_{d}\right)$ as:

(1) $$n_{eff}\left(n_{d}\right)=n_{eff}^{r}\left(n_{d}\right)+in_{eff}^{i}\left(n_{d}\right)$$

(2) $$\alpha \left(n_{d}\right)=k_{0}n_{eff}^{i}\left(n_{d}\right)\;\;\;;\;\;\;k_{0}=\omega /c$$

Transmission losses can be obtained by monitoring intensity of the transmitted light $I_{out}$ at the sensor output end as:

(3) $$I_{out}\left(n_{d}+\delta n_{d}\right)=I_{in}e^{-2\alpha \left(n_{d}+\delta n_{d}\right)L}$$

where $I_{in}$ is the light intensity at the sensor input end, and *L* is the sensor length. Thus, by comparing light intensities before $I_{out}\left(n_{d}\right)$ and after $I_{out}\left(n_{d}+\delta n_{d}\right)$ changes in the analyte refractive index took place, from Equation (3) we can write:

(4) $$\frac{I_{out}\left(n_{d}+\delta n_{d}\right)}{I_{out}\left(n_{d}\right)}=\exp\left(-2\frac{\partial \alpha \left(n_{d}\right)}{\partial n_{d}}L\cdot \delta n_{d}\right)=e^{-S^{A}\delta n_{d}}$$

where parameter $S^{A}$ characterizes the amplitude sensitivity and is proportional to the sensor length. Recognizing that the maximal sensor length $L_{max}$ is limited by the propagation loss of the surface mode:

(5) $$L_{max}\sim \frac{1}{\alpha \left(n_{d}\right)}$$

We can estimate the maximal value of the amplitude sensitivity and relate it to the modal refractive index (using Equations (1) and (2) as:

(6) $$S_{max}^{A}\left[\frac{1}{RIU}\right]=2\frac{\partial \alpha \left(n_{d}\right)}{\partial n_{d}}L_{max}\sim \frac{2}{n_{eff}^{i}\left(n_{d}\right)}\frac{\partial n_{eff}^{i}\left(n_{d}\right)}{\partial n_{d}}$$

Finally, Equation (4) also allows us to estimate sensor resolution. Particularly, defining ${\left(\delta I/I\right)}_{min}$ to be the smallest reliably detectable difference in the relative amplitude (which is typically smaller than 0.01), from Equation (4) it follows that the smallest detectable change in the analyte refractive index will be:

(7) $${\left(\delta n_{d}\right)}_{min}^{A}\sim \frac{{\left(\delta I/I\right)}_{min}}{S_{max}^{A}}$$

### 2.2. Phase Sensing Modality

**Figure 2:**
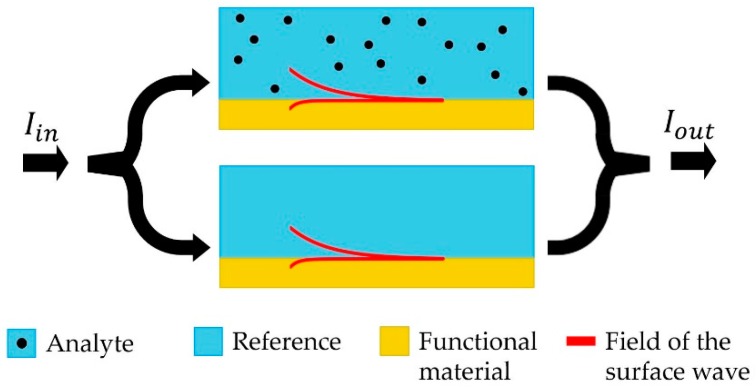
Schematic of a phase sensitive detection strategy. Coherent light is subdivided in two beams, one propagating through the reference and another through a sensor. The two beams are then recombined and resulting interference pattern is used to monitor variation in the analyte refractive index.

Phase sensing modality, compared to amplitude-based strategy, offers a considerably more precise and better controlled technique for monitoring changes in the analyte refractive index. A simplified schematic of the phase-based sensor is presented in Figure 2. In such a sensor one frequently uses a Mach–Zehnder interferometer to convert a relative phase difference of light traveling along the reference and sensing pathways into an easily detectable amplitude variation. Phase sensing detection modality is, therefore, mostly used to detect variations in the real part of the analyte refractive index.

To introduce the phase sensitivity parameter, we have to analyze amplitude of the detected signal at the output of a Mach-Zehnder interferometer shown in Figure 2. Particularly, the electric fields after propagation along the length L in the reference and sensing branches of the interferometer are given by:

(8) $$E\left(n_{d},\;L\right)=E_{in}e^{ik_{0}n_{eff}\left(n_{d}\right)L}$$

(9) $$\;E\left(n_{d}+\delta n_{d},\;L\right)=E_{in}e^{ik_{0}n_{eff}\left(n_{d}+\delta n_{d}\right)L}\;$$

Assuming that the two branches show similar losses ($\alpha \left(n_{d}\right)\approx \alpha \left(n_{d}+\delta n_{d}\right)$), then the interference of light coming from the two branches will result in the following output intensity:

(10) $$\begin{array}{l} I_{out}\left(n_{d}+\delta n_{d}\right)={\left|E\left(n_{d}\right)+E\left(n_{d}+\delta n_{d}\right)\right|}^{2}\\ \;\;\;\;\;\;=4E_{in}^{2}\exp\left(-2\alpha \left(n_{d}\right)L\right)\cos\left(\frac{k_{0}L}{2}\frac{\partial n_{eff}^{r}\left(n_{d}\right)}{\partial n_{d}}\delta n_{d}\right)\\ \;\;\;\;\;\;=4E_{in}^{2}\exp\left(-2\alpha \left(n_{d}\right)L\right)\cos\left(S^{P}\delta n_{d}\right) \end{array}$$

Here, parameter $S^{P}$ characterizes the phase sensitivity and is proportional to the sensor length. Recognizing that the maximal sensor length $L_{max}$ is limited by the propagation loss of the surface mode $L_{max}\sim 1/\alpha \left(n_{d}\right)$, and using Equation (2), the maximal phase sensitivity can be estimated as:

(11) $$S_{max}^{P}\left[\frac{rad}{RIU}\right]=\frac{k_{0}L_{max}}{2}\frac{\partial n_{eff}^{r}\left(n_{d}\right)}{\partial n_{d}}\sim \frac{1}{2n_{eff}^{i}\left(n_{d}\right)}\frac{\partial n_{eff}^{r}\left(n_{d}\right)}{\partial n_{d}}$$

Finally, Equation (10) also allows us to estimate sensor resolution. Particularly, defining $\delta \varphi_{min}$ to be the smallest reliably detectable variation in the phase of an output signal (which is normally much smaller than π), from Equation (10) it follows that the smallest detectable change in the analyte refractive index will be:

(12) $${\left(\delta n_{d}\right)}_{min}^{P}\sim \frac{\delta \varphi_{min}}{S_{max}^{P}}$$

## 3. Theory of Surface Waves in the Context of Sensing Applications

In what follows, we analyze the performance characteristics and application scenarios of the amplitude and phase sensing techniques described in the previous sections in the context of surface waves propagating at the interface between a functional material and an analyte (see Figure 1 and Figure 2). Particularly, we aim at understanding the fundamental relations between the sensor sensitivity, the extent of the modal field of a surface wave into the analyte, and the maximal propagation length of a surface wave. Also, we consider a sensor to be a “surface sensor” if the modal field extent of a surface wave into the analyte (probing depth) is subwavelength or at most several wavelengths, while we consider a sensor to be a “bulk sensor” if the modal field extent of a surface wave into the analyte is many wavelengths. Remarkably, we find that in the case of low-loss analytes, and for many types of surface waves, sensor sensitivity correlates strongly with the modal probing depth. Thus, bulk sensors characterized by long probing depths tend to have higher sensitivities than the surface sensors characterized by subwavelength probing depths.

### 3.1. Dispersion Relation of Surface Waves

Interaction between electromagnetic (E and M) waves and free charge carriers can yield surface plasmon polaritons (SPP) excitation at the metal or semiconductor/dielectric interfaces, while interaction of E and M waves with lattice vibrational modes lead to surface phonon polaritons at the polar media/dielectric interface. Moreover, well-confined surface-bound E and M states can be excited at the interface with a high-k dielectric having high material losses, which are known as a Zenneck surface waves. Finally, surface waves can be excited at the interface with a naturally-occurring or artificially-structured anisotropic media, in which case such waves are known as Dyakonov surface waves. Each one of these surface waves has its advantages and limitations and requires a particular set of material properties for their excitation. Moreover, due to variations of the material properties with frequency, such surface waves can have completely different optical properties depending on the frequency range of operation. In this work we focus specifically on surface waves in the THz spectral range.

**Figure 3:**
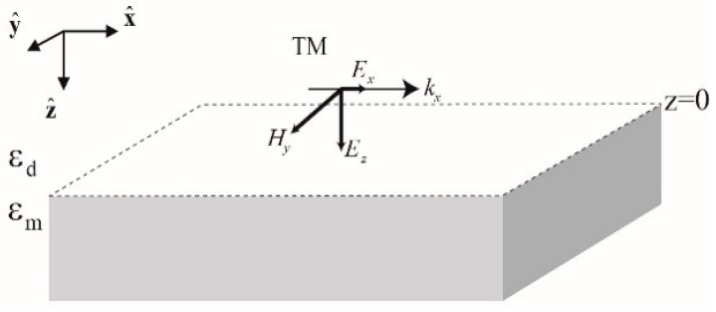
Schematic of an interface between two materials that guides a TM polarized surface wave.

We first review the necessary conditions for the excitation of surface waves at the interface between two distinct materials, one of them being lossless dielectric with positive dielectric permittivity $\varepsilon_{d}$ and another material with dielectric permittivity $\varepsilon_{m}$ that can have either negative real part (metal, semiconductor, polar media just above the resonance) or positive real part (polar media just below the resonance, high-k dielectric). Figure 3 shows planar interface between two materials (OXY plane), and a TM-polarized electromagnetic (E and M) wave that has the only component of its magnetic field directed along the OY axis.

On either side of the interface we assume a single TM polarized planewave of frequency ω and the corresponding E and M wavevectors $k_{d,m}=\left(k_{x},0,k_{z}^{d,m}\right)$. The corresponding magnetic and electric fields on either side of the interface are given by:

(13) $$H_{d,m}\left(r,t\right)=\hat{y}H_{d,m}e^{ik_{d,m}r-i\omega t}$$

(14) $$\;E_{d,m}\left(r,t\right)=\frac{H_{d,m}\left(r,t\right)\times k_{d,m}}{\varepsilon_{d,m}k_{0}},$$

where $k_{0}=\omega /c=2\pi /\lambda$, and the wavevectors satisfy the standard dispersion relations:

(15) $$k_{d,m}^{2}=\varepsilon_{d,m}k_{0}^{2}=k_{x}^{2}+{\left(k_{z}^{d,m}\right)}^{2}$$

The E and M field components parallel to the interface must be continuous across the interface ($\hat{y}H_{d}\left(0,t\right)=\hat{y}H_{m}\left(0,t\right);\hat{x}E_{d}\left(0,t\right)=\hat{x}E_{m}\left(0,t\right)$), thus leading to the following equation for the $k_{z}^{\left(d,m\right)}$:

(16) $$\frac{k_{z}^{d}}{\varepsilon_{d}}-\frac{k_{z}^{m}}{\varepsilon_{m}}=0$$

Using dispersion relations (Equation (15), as well as definition of the modal effective refractive index $k_{x}=n_{eff}k_{0}$, Equation (16) admits a simple analytical solution for the surface mode dispersion relation:

(17) $$\varepsilon_{eff}=n_{eff}^{2}=\frac{\varepsilon_{d}\varepsilon_{m}}{\varepsilon_{d}+\varepsilon_{m}};{\left(k_{z}^{d,m}\right)}^{2}=k_{0}^{2}\frac{{\left(\varepsilon_{d,m}\right)}^{2}}{\varepsilon_{d}+\varepsilon_{m}}$$

While Equation (17) is a solution of Equation (16), it does not necessarily describe a bound surface state as Equation (16) is just a condition of E and M field continuity across the interface. In order for Equation (17) to describe a surface state, one must require that the electromagnetic fields are decaying away from the interface (evanescent fields), which amounts to additional requirements:

(18) Im(kzd)<0   ;  Im(kzm)>0

and define exponentially decaying fields in both media. Finally, for a surface-bound state we define its propagation length $L_{x}$ as well as its extents into a dielectric $L_{d}$ and a second material $L_{m}$ as characteristic distances over which the E and M fields decay by the factor *e*:

(19) Lx=1Im(kx)   ;   Ld,m=1∓Im(kzd,m)

Now we detail several types of surface states propagating at the interface between a classic lossless dielectric with $\varepsilon_{d}>0$ and a second material that can be either a metal, a polar material, a zero-gap material, or a lossy high-k dielectric. Particularly, in the case of simple Drude metals and semiconductors, their frequency-dependent dielectric constant is described as [9]:

(20) $$\varepsilon_{m}\left(\omega \right)=1-\frac{\omega_{p}^{2}}{\omega^{2}+i\gamma \omega }$$

where $\omega_{p}$ is a plasma frequency and γ is a damping coefficient. Similarly, the dielectric constant of many polar materials near the resonance frequency $\omega_{0}$ can be described using the Lorentz model:

(21) $$\varepsilon_{m}\left(\omega \right)=1-\frac{\omega_{p}^{2}}{\omega^{2}-\omega_{0}+i\gamma \omega }$$

while lossy high-k dielectrics are characterized by $\varepsilon_{m}=\varepsilon_{m}^{\prime }+i\varepsilon_{m}^{\prime \prime }$, where $\varepsilon_{m}^{\prime }\gg 1$.

### 3.2. Lossless Materials

We first consider the case of ideal (lossless) materials γ=0 operating at frequencies where their dielectric constant is negative so that the two following conditions are satisfied $\varepsilon_{m}<0$ and $\varepsilon_{d}+\varepsilon_{m}<0$. In this case, the surface mode effective dielectric constant as given by (17) is purely real and positive $\varepsilon_{eff}>\varepsilon_{d}$. For metals and polar materials, this limits the operation frequency to:

(22) $$\mathrm{Metals}:\omega <\frac{\omega_{p}}{\sqrt{1+\varepsilon_{d}}}\;\;\;;\;\;\;\mathrm{Polar}\;\mathrm{materials}:\;\omega_{0}<\omega <\sqrt{\omega_{0}^{2}+\frac{\omega_{p}^{2}}{1+\varepsilon_{d}}}$$

At such frequencies, the OZ components of the two wavevectors are purely imaginary and their imaginary parts have the opposite signs:

(23) $$k_{z}^{d,m}=\mp ik_{0}\frac{\left|\varepsilon_{d,m}\right|}{\sqrt{\left|\varepsilon_{m}\right|-\varepsilon_{d}}}$$

thus defining a true surface state, which is called a surface plasmon polariton in case of metals and surface phonon polariton in case of polar materials. Electromagnetic fields of such a surface state decay exponentially fast away from the interface. The surface mode propagation length $L_{x}$ is infinite as $n_{eff}$ is purely real, while the surface mode extents into a dielectric $L_{d}$ and a material $L_{m}$ are given by Equation (24):

(24) $$L_{d,m}=\frac{\lambda }{2\pi }\frac{\sqrt{\left|\varepsilon_{m}\right|-\varepsilon_{d}}}{\left|\varepsilon_{d,m}\right|}$$

Note that plasmon polariton or phonon polariton extents into the two media become deeply subwavelength $L_{d,m}\ll \lambda$ when $\left|\varepsilon_{m}\right|\sim \varepsilon_{d}$, which happens near the following frequencies:

(25) $$\mathrm{Metals}:\omega ∼\frac{\omega_{p}}{\sqrt{1+\varepsilon_{d}}}\;\;\;;\;\;\;\mathrm{Polar}\;\mathrm{materials}:\;\omega ∼\sqrt{\omega_{0}^{2}+\frac{\omega_{p}^{2}}{1+\varepsilon_{d}}}$$

**Figure 4:**
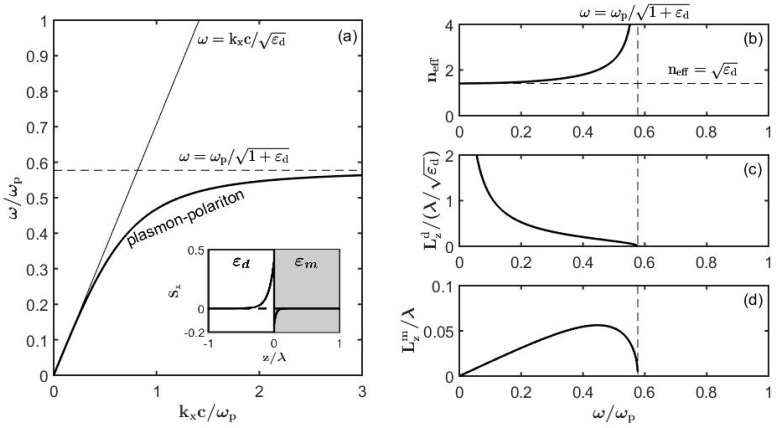
Dispersion relation and fundamental properties of a lossless surface plasmon polaritons ($n_{d}=1.33$). (**a**) Band diagram of a surface plasmon. Insert: $S_{x}$ energy flux of a plasmon at $\omega =0.4\omega_{p}$. Optical properties of a plasmon: (**b**) Effective refractive index, (**c**) penetration depth into the dielectric, and (**d**) penetration depth into metal.

In Figure 4 we present a typical lossless surface plasmon polariton dispersion relation as well as dependence of its penetration depths into a dielectric and metal as a function of frequency. The surface plasmon-polariton extent into metal is always deeply subwavelength $L_{z}^{m}\ll \lambda$, while its extent into the dielectric is generally comparable or even smaller than the wavelength of light $L_{z}^{d}<\lambda$ at higher frequencies.

**Figure 5:**
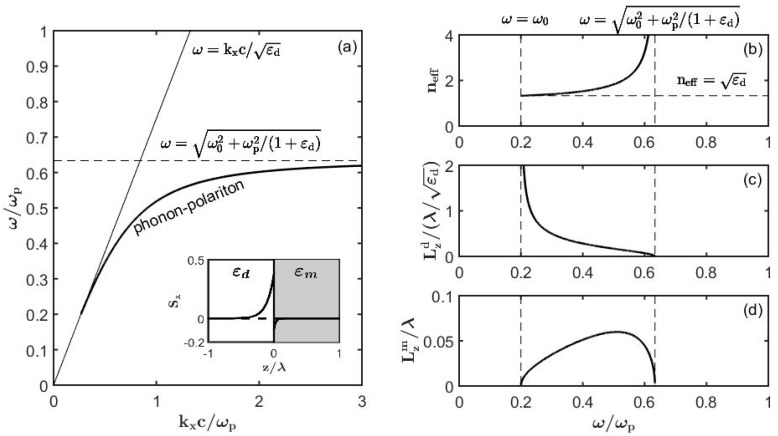
Dispersion relation and fundamental properties of a lossless surface phonon polariton ($n_{d}=1.33,\omega_{0}=0.2\omega_{p}$). (**a**) Band diagram of a surface phonon polariton. Insert: $S_{x}$ energy flux of a phonon polariton at $\omega =0.4\omega_{p}$. Optical properties of a phonon polariton: (**b**) Effective refractive index, (**c**) penetration depth into the dielectric, and (**d**) penetration depth into the polar medium.

In Figure 5 we present similar results for a surface phonon polariton, and arrive to a similar conclusion that somewhat above the resonant frequency $\omega_{0}$, the surface phonon-polariton extent into a polar material is always deeply subwavelength $L_{z}^{m}\ll \lambda$, while its extent into the dielectric is generally comparable, or even smaller than the wavelength of light $L_{z}^{d}<\lambda$ at higher frequencies.

### 3.3. Lossy Materials

When material losses are small:

(26) $$\mathrm{Metals}:\gamma \ll \frac{\omega_{p}}{\sqrt{1+\varepsilon_{d}}}\;\;\;;\;\;\;\mathrm{Polar}\;\mathrm{materials}:\gamma \ll \sqrt{\omega_{0}^{2}+\frac{\omega_{p}^{2}}{1+\varepsilon_{d}}}-\omega_{0}$$

Conclusions drawn in the case of lossless materials will still hold virtually unchanged, while corrections to the modal optical properties could be found using perturbative expansions with respect to the imaginary part of the dielectric constant. In some cases, however, material losses cannot be ignored as imaginary part of the material dielectric constant can be comparable or larger than its real part. This is notably the case of Drude metals at very low frequencies ω≪γ operating in the THz and microwave spectral ranges. At such frequencies, the metal dielectric constant is essentially purely imaginary. Remarkably, even at very low frequencies a well-defined surface state with complex dispersion (Equation (17) still exists, as we will see in the following sections.

### 3.4. Lossy High-k Dielectrics

A lossy surface-bound E and M state (also known as a Zenneck wave) can be excited at the interface between two distinct dielectrics both having positive real parts of the dielectric constants. In what follows we assume that one dielectric is lossless $\varepsilon_{d}>0$, while another is a high-k lossy dielectric $\varepsilon_{m}=\varepsilon_{m}^{\prime }+i\varepsilon_{m}^{\prime \prime }$, where, additionally, $\varepsilon_{m}^{\prime }\gg \varepsilon_{d}$. One can then directly use the expressions for the modal effective refractive index (17) and modal extents into the dielectric materials (Equation (19), while choosing the signs of the transverse wavevectors in both media to satisfy the surface-bounded wave condition (Equation (18). Resultant expressions can be further simplified in the limit $\varepsilon_{m}^{\prime \prime }\ll \varepsilon_{m}^{\prime }$, which is the case for most high-k dielectrics to obtain the following expressions for the modal propagation distance (limited by material losses), and modal extents into the two dielectrics:

(27) $$n_{eff}\underset{\varepsilon_{m}^{\prime \prime }\ll \varepsilon_{m}^{\prime }}{\approx }\sqrt{\frac{\varepsilon_{d}\varepsilon_{m}^{\prime }}{\varepsilon_{d}+\varepsilon_{m}^{\prime }}}\left(1+i\frac{\varepsilon_{d}\varepsilon_{m}^{\prime \prime }}{2\varepsilon_{m}^{\prime }\left(\varepsilon_{d}+\varepsilon_{m}^{\prime }\right)}\right)\underset{\varepsilon_{d}\ll \varepsilon_{m}^{\prime }}{\approx }n_{d}-\frac{n_{d}^{3}}{2\varepsilon_{m}^{\prime }}+i\frac{n_{d}^{3}\varepsilon_{m}^{\prime \prime }}{2{\left(\varepsilon_{m}^{\prime }\right)}^{2}}$$

(28) $$\;L_{d}\underset{\varepsilon_{m}^{\prime \prime }\ll \varepsilon_{m}^{\prime }}{\approx }\frac{\lambda }{\pi }\frac{\sqrt{\varepsilon_{d}}}{\varepsilon_{m}^{\prime \prime }}{\left(1+\frac{\varepsilon_{m}^{\prime }}{\varepsilon_{d}}\right)}^{\frac{3}{2}}\underset{\varepsilon_{d}\ll \varepsilon_{m}^{\prime }}{\approx }\frac{\lambda }{\pi }\frac{{\left(\varepsilon_{m}^{\prime }\right)}^{\frac{3}{2}}}{\varepsilon_{m}^{\prime \prime }\varepsilon_{d}}\;$$

(29) $$\;L_{m}\underset{\varepsilon_{m}^{\prime \prime }\ll \varepsilon_{m}^{\prime }}{\approx }\frac{L_{d}}{2+\varepsilon_{m}^{\prime }/\varepsilon_{d}}\underset{\varepsilon_{d}\ll \varepsilon_{m}^{\prime }}{\approx }L_{d}\frac{\varepsilon_{d}}{\varepsilon_{m}^{\prime }}\ll L_{d}\;$$

(30) $$\;L_{x}\underset{\varepsilon_{m}^{\prime \prime }\ll \varepsilon_{m}^{\prime }}{\approx }L_{d}\sqrt{\frac{\varepsilon_{m}^{\prime }}{\varepsilon_{d}}}\gg L_{d}\;$$

From these expressions we note that, in the case of high-k dielectrics, $\varepsilon_{m}^{\prime }\gg \varepsilon_{d}$, the Zenneck wave is well-defined as its propagation distance is much larger than the modal extents into both dielectrics $L_{x}\gg L_{m,d}$. In this limit Zenneck waves are also known as Brewster waves [10]. At the same time, modal extent into the high-k dielectric is much smaller than that into the lossless dielectric $L_{m}\ll L_{d}$. At the same time, achieving strong confinement of Zenneck waves near the surface is somewhat problematic due to inverse dependence of its transverse size on the loss of a high-k dielectric. Nevertheless, as it follows from the further analysis of (28), (17) and (19) by using high-k dielectrics with high material losses Im(εm)~Re(εm) allows modal size *L_d_* in the 2λ−10λ range, thus making lossy high-k dielectrics viable candidates for surface wave excitation.

### 3.5. Summary of Surface Waves and Their Classification

**Figure 6:**
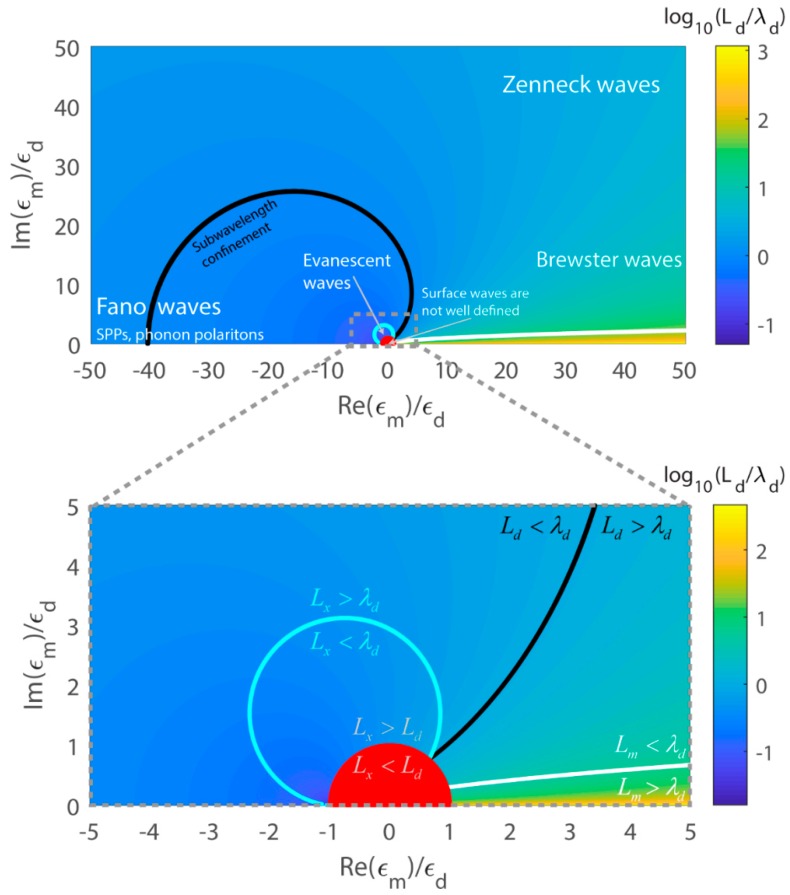
Classification of the surface waves and summary of their optical properties as a function of the complex value of the material dielectric constant (Re(εm)/εd,Im(εm)/εd), assuming that the bordering dielectric (analyte) has a purely real positive dielectric constant $\varepsilon_{d}$. Plotted is the relative value of the surface wave probing depth into the dielectric (analyte), $L_{d}/\lambda_{d}$, where $\lambda_{d}$ is the wavelength of light in the material, $\lambda_{d}=\lambda /\sqrt{\varepsilon_{d}}$. The plot is universal in these normalised coordinates.

Here we summarise the optical properties and classify the surface waves presented so far (classification and naming convention follows that of [10]). To simplify presentation, we define $\lambda_{d}=\lambda /\sqrt{\varepsilon_{d}}$ to be the wavelength of light in the lossless dielectric. In Figure 6 we plot the value of the surface wave relative extent into dielectric $L_{d}/\lambda_{d}$ calculated using Equations (17)–(19) as a function of the normalized real and imaginary parts of the material dielectric constant (Re(εm)/εd,Im(εm)/εd).

We first note that for any choice of the material dielectric constant $\varepsilon_{m}$ one can show that the surface wave penetration into material is always smaller than the surface wave penetration into dielectric $L_{m}<L_{d}$. Furthermore, in Figure 6 we show a black solid line that separates the regime of subwavelength confinement $L_{d}<\lambda_{d}$ from the regime of super-wavelength confinement $L_{d}>\lambda_{d}\;$; here we note that subwavelength confinement is achieved within a certain bounded region of absolute values of the material dielectric constant $1<\left|\varepsilon_{m}/\varepsilon_{d}\right|<50$. Additionally, inside a red region near the origin (dielectric constant values $\left|\varepsilon_{m}/\varepsilon_{d}\right|<1$) surface waves are not well defined as their propagation distance along the surface becomes smaller than their extent into dielectric $L_{x}<L_{d}$, while outside of the red zone, surface waves are well-defined in the sense $L_{x}>L_{d}$.

Now we consider the regime of Re(εm)<−εd and Im(εm)≪Re(εm) where Fano-type surface waves can be excited that correspond to classical surface plasmon polaritons and surface phonon polaritons. Surface plasmon polaritons of this type are typically observed in the visible and near-IR spectral range using simple metals, while at lower frequencies (THz, microwaves) one has to resort to semiconductors to excite this type of a wave. Surface phonon polaritons of this type can be observed in the mid-IR and lower frequencies (THz, microwaves) using various types of polaritonic (ex. ferroelectrics) materials. In this regime both sub- and super-wavelength probing depths into dielectric are possible depending on the value of the negative real part of the material dielectric constant. In this regime, the probing depth into the material is deeply subwavelength, $L_{m}\ll \lambda_{d}$.

Next, we consider the regime of Re(εm)>0 and Im(εm)≫Re(εm) where Zenneck-type surface waves can be excited. Many practical materials that support Zenneck waves also feature high conductivities so that Im(εm)≫εd. In this regime the probing depth into dielectric of Zenneck waves is typically significantly super-wavelength, $L_{d}\gg \lambda_{d}$, while their penetration into the material is deeply subwavelength, $L_{m}\ll \lambda_{d}$. In fact, from Figure 6 we note that from the point of view of surface wave confinement, there is no conceptual difference between classic Zenneck waves and the waves excited on materials with negative value of the dielectric constant Re(εm)<0, as long as Im(εm)≫Re(εm), and Im(εm)>30. This type of surface waves can be observed at lower frequencies (THz, microwaves, radio waves), for example, in materials featuring high electronic conductivity (simple metals in THz and microwaves, Earth as a conductive ground for radio waves).

Then, we consider the regime of Re(εm)>0 and 0<Im(εm)≪Re(εm) where Brewster-type surface waves can be excited. In this regime surface wave probing depth into dielectric can become super-wavelength, $L_{m}>\lambda_{d}$ (located below white solid line in Figure 6). Additionally, in the case of lossy high-k dielectrics featuring Re(εm)≫εd, Im(εm)≫εd, the probing depths into the dielectric are somewhat super-wavelength, $L_{d}>\lambda_{d}$, while their penetration into the material are weakly subwavelength, $L_{m}<\lambda_{d}$.

Finally, another interesting regime (located within a cyan circle in Figure 6) is near the origin where evanescent waves can be excited. These surface waves are well defined as their propagation length can be much longer than their extent into the dielectric, $L_{x}>L_{d}$, while at the same time both the modal extent into dielectric, as well as modal propagation length, are subwavelengths $L_{d}\ll \lambda_{d}$, $L_{x}<\lambda_{d}$.

## 4. Sensor Sensitivities

We now derive expressions for the sensor sensitivities for the amplitude and phase sensor modalities when using various types of surfaces waves. For completeness, we also present expressions for the modal probing depths into dielectric $L_{d}$, as well as modal propagation length $L_{x}$.

### 4.1. Plasmon Polaritons on Simple Metals

In the THz spectral range for most simple (Drude) metals ω≪γ, and their dielectric constant (Equation (20)) is almost purely imaginary $\varepsilon_{m}^{\prime \prime }\gg \left|\varepsilon_{m}^{\prime }\right|$, thus:

(31) $$\varepsilon_{m}\left(\omega \right)=\varepsilon_{m}^{\prime }+i\varepsilon_{m}^{\prime \prime }\underset{\omega \ll \gamma }{\approx }-\frac{\omega_{p}^{2}}{\gamma^{2}}+i\frac{\omega_{p}^{2}}{\gamma \omega }$$

Additionally, for most analytes in the THz spectral range $\varepsilon_{d}\ll \left|\varepsilon_{m}^{\prime }\right|$ and, in this limit, the effective refractive index of the surface wave given by Equation (17) becomes:

(32) $$n_{eff}\left(n_{d}\right)\underset{\varepsilon_{m}^{\prime \prime }\gg \left|\varepsilon_{m}^{\prime }\right|\gg \varepsilon_{d}}{\approx }n_{d}-\frac{n_{d}^{3}\varepsilon_{m}^{\prime }}{2{\left(\varepsilon_{m}^{\prime \prime }\right)}^{2}}+i\frac{n_{d}^{3}}{2\varepsilon_{m}^{\prime \prime }}$$

while modal probing depth into the dielectric and modal propagation length are:

(33) $$L_{d}\underset{\varepsilon_{m}^{\prime \prime }\gg \left|\varepsilon_{m}^{\prime }\right|\gg \varepsilon_{d}}{\approx }\frac{\lambda }{\sqrt{2}\pi }\frac{\sqrt{\varepsilon_{m}^{\prime \prime }}}{\varepsilon_{d}}$$

(34) $$\;L_{x}\underset{\varepsilon_{m}^{\prime \prime }\gg \left|\varepsilon_{m}^{\prime }\right|\gg \varepsilon_{d}}{\approx }\frac{\lambda }{\pi }\frac{\varepsilon_{m}^{\prime \prime }}{n_{d}^{3}}\;$$

Then, by substituting Equation (32) into the expressions for the amplitude (Equation (6)) and phase (Equation (11)) sensitivities we get for plasmon polariton waves on simple metals in the THz spectral range:

(35) $$S^{A}\left[\frac{1}{RIU}\right]\underset{\varepsilon_{m}^{\prime \prime }\gg \left|\varepsilon_{m}^{\prime }\right|\gg \varepsilon_{d}}{\approx }\frac{6}{n_{d}}$$

(36) $$\;S^{P}\left[\frac{rad}{RIU}\right]\underset{\varepsilon_{m}^{\prime \prime }\gg \left|\varepsilon_{m}^{\prime }\right|\gg \varepsilon_{d}}{\approx }\frac{\varepsilon_{m}^{\prime \prime }}{n_{d}^{3}}\approx \pi \left(\frac{L_{x}}{\lambda }\right)\approx 2\pi^{2}n_{d}{\left(\frac{L_{d}}{\lambda }\right)}^{2}\;$$

We note that while amplitude sensitivity of the THz surface plasmon-polariton sensors is mostly frequency independent, their phase sensitivity is strongly frequency dependent and scale proportionally to the square of the wave probing depth $L_{d}$ into the analyte.

### 4.2. Phonon-Polaritons on Polar Materials, and Plasmon Polaritons on Semiconductors and Zero-Gap Materials

In addition to simple metals, plasmon-polaritons can also be excited at the interface with a semiconductor or a zero-gap material, while phonon-polariton can be excited at the interface with a polar material as long as $\varepsilon_{m}^{\prime }<0$, and $\left|\varepsilon_{m}^{\prime }\right|>\varepsilon_{d}$. In this general case, material dielectric constant is often characterized by mostly real negative dielectric constant $\varepsilon_{m}^{\prime \prime }\ll \left|\varepsilon_{m}^{\prime }\right|-\varepsilon_{d}$, and in this limit the effective refractive of a surface wave is given by:

(37) $$n_{eff}\left(n_{d}\right)\underset{\varepsilon_{m}^{\prime \prime }\gg \left|\varepsilon_{m}^{\prime }\right|\gg \varepsilon_{d}}{\approx }n_{d}-\frac{n_{d}^{3}\varepsilon_{m}^{\prime }}{2{\left(\varepsilon_{m}^{\prime \prime }\right)}^{2}}+i\frac{n_{d}^{3}}{2\varepsilon_{m}^{\prime \prime }}$$

while modal probing depth into the dielectric and modal propagation length are:

(38) $$L_{d}\underset{\varepsilon_{m}^{\prime \prime }\ll \left|\varepsilon_{m}^{\prime }\right|-\varepsilon_{d}}{\approx }\frac{\lambda }{2\pi n_{d}}{\left(\frac{\left|\varepsilon_{m}^{\prime }\right|}{\varepsilon_{d}}-1\right)}^{\frac{1}{2}}$$

(39) $$L_{x}\underset{\varepsilon_{m}^{\prime \prime }\ll \left|\varepsilon_{m}^{\prime }\right|-\varepsilon_{d}}{\approx }\frac{\lambda }{\pi }\frac{\sqrt{\left|\varepsilon_{m}^{\prime }\right|}}{\varepsilon_{m}^{\prime \prime }}{\left(\frac{\left|\varepsilon_{m}^{\prime }\right|}{\varepsilon_{d}}-1\right)}^{\frac{3}{2}}$$

and the sensitivities for the two sensor modalities are then given by:

(40) $$S^{A}\left[\frac{1}{RIU}\right]\underset{\varepsilon_{m}^{\prime \prime }\ll \left|\varepsilon_{m}^{\prime }\right|-\varepsilon_{d}}{\approx }\frac{6}{n_{d}}\frac{\left|\varepsilon_{m}^{\prime }\right|}{\left|\varepsilon_{m}^{\prime }\right|-\varepsilon_{d}}$$

(41) $$\;S^{P}\left[\frac{rad}{RIU}\right]\underset{\varepsilon_{m}^{\prime \prime }\ll \left|\varepsilon_{m}^{\prime }\right|-\varepsilon_{d}}{\approx }\frac{1}{n_{d}^{3}}\frac{{\left(\varepsilon_{m}^{\prime }\right)}^{2}}{\varepsilon_{m}^{\prime \prime }}\;$$

We note that both amplitude and phase sensitivities of thus described THz surface plasmon-polariton and surface phonon-polariton sensors can be strongly frequency dependent, especially in the vicinity of plasmon/phonon-polariton cutoff frequencies at which $\varepsilon_{m}^{\prime }+\varepsilon_{d}=0$.

### 4.3. Zenneck Waves

Finally, at the interface with a high-k dielectric ($\varepsilon_{m}^{\prime }>0$) for which $\varepsilon_{m}^{\prime \prime }\ll \varepsilon_{m}^{\prime }$, we use expressions presented in Section 3.4 for the Zenneck wave effective refractive index, modal probing depth into the dielectric, and modal propagation length to obtain the following sensitivities for the two sensor modalities:

(42) $$S^{A}\left[\frac{1}{RIU}\right]\underset{\varepsilon_{m}^{\prime \prime }\ll \varepsilon_{m}^{\prime }}{\approx }\frac{6}{n_{d}}\frac{\varepsilon_{m}^{\prime }}{\varepsilon_{m}^{\prime }+\varepsilon_{d}}\underset{\varepsilon_{d}\ll \varepsilon_{m}^{\prime }}{\approx }\frac{6}{n_{d}}$$

(43) $$\;S^{P}\left[\frac{rad}{RIU}\right]\underset{\varepsilon_{m}^{\prime \prime }\ll \varepsilon_{m}^{\prime }}{\approx }\frac{1}{n_{d}^{3}}\frac{{\left(\varepsilon_{m}^{\prime }\right)}^{2}}{\varepsilon_{m}^{\prime \prime }}\underset{\varepsilon_{d}\ll \varepsilon_{m}^{\prime }}{\approx }\pi \left(\frac{L_{x}}{\lambda }\right)\propto \left(\frac{L_{d}}{\lambda }\right)\;$$

We note that while amplitude sensitivity of the THz Zenneck wave on high-k dielectric sensors is mostly frequency independent, their phase sensitivity can be strongly frequency dependent and generally scale proportionally to the wave probing depth $L_{d}$ into the analyte (as both $S^{P}$ and $L_{d}$ are proportional to $\varepsilon_{m}^{\prime }/\varepsilon_{m}^{\prime \prime }$).

## 5. Review of Materials, Corresponding Surface Waves, and Sensor Performance

In what follows, we review major classes of materials that support surface waves at THz frequencies and study their potential for sensing applications. We concentrate mostly on the uniform homogeneous materials which are the simplest to process and shape into desired devices, while only mentioning artificially structured materials in passing.

### 5.1. Simple Metals

**Figure 7:**
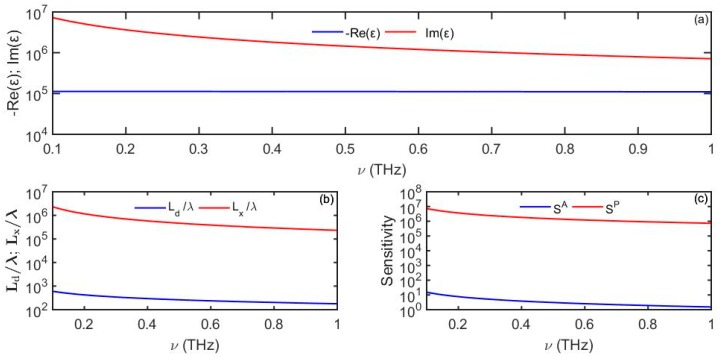
(**a**) Complex dielectric function of gold following the Drude model. (**b**) Plasmon penetration depth into the analyte and propagation distance. (**c**) Amplitude-based sensitivity *S^A^* (1/RIU) and phase-based sensitivity *S^P^* (rad/RIU) as a function of frequency. For the analyte we use *n_d_* = 1.

First, we consider simple metals which are mainly used in the fabrication of SPR sensors operating in the visible and IR spectral ranges [11]. As an example, in Figure 7 we plot properties of a surface plasmon polariton propagating at the gold/gaseous analyte ($n_{d}=1$) interface in the THz spectral range and observe that while the surface state shows very low loss (long propagation distances) $L_{x}\gg \lambda$, at the same time, it is highly delocalized into the dielectric material $L_{d}\gg \lambda$. In fact, the probing depth of a surface wave into gaseous analyte can be as high as ∼10 cm, thus making such sensors sensitive to the “bulk” changes in the analyte refractive index. Here, we used $\omega_{p}\;=2\pi \times 2175$ THz and γ=2π×6.48 THz for gold [9].

We observe that in theory a phase-based sensor using gold as a functional material can be very sensitive to changes in the refractive index of analyte mainly due to low-loss of a surface plasmon, and as a consequence, very long sensor lengths. However, to achieve such high sensitivities as predicted by Equation (36), THz plasmon-polaritons must propagate along the gold-dielectric interface for up to the theoretical propagation length of ∼10–100 m. It is important to note that the experimental values of the plasmon propagation lengths along planar gold interfaces are in fact few orders of magnitude smaller than the predicted theoretical values based on the Drude model [12]. This is to be expected as the model mostly describes bulk optical properties of simple metals, while conductive surfaces might demonstrate additional phenomena not captured by the Drude model [13,14,15,16], such as surface structure modification due to atomic surface reconstruction, oxidation, pollutants, etc., as well as additional scattering losses due to geometrical imperfections at the metal surfaces, such as roughness, clustering, etc. These effects tend to increase scattering loses at the metal surface, thus leading to smaller plasmon propagation lengths and lower sensitivities of sensors based on SPPs.

### 5.2. Semiconductors

Next, we consider semiconductor materials which are directly analogous to simple metals and can often be described using a Drude model (Equation (20)). However, plasma frequency of semiconductor materials generally expressed as:

(44) $$\omega_{p}=\sqrt{\frac{e^{2}N}{\varepsilon_{0}m^{*}}}$$

is generally significantly lower than that of the simple metals (and can even be pushed into the THz spectral range) due to lower concentrations of the free carriers N. We are particularly interested in materials that have plasma frequency in the THz spectral range, while also having relatively small absorption loss so that $\varepsilon_{m}^{\prime }+\varepsilon_{d}<0$ and $\varepsilon_{m}^{\prime \prime }\ll \left|\varepsilon_{m}^{\prime }\right|-\varepsilon_{d}$. Under these conditions (see Section 4.2), such materials can support tightly localized surface states in the THz band that are directly analogous to plasmon polaritons in the visible spectral range.

As an example, consider InSb, a semiconductor with plasma frequency of 7.32 THz that can support highly localized surface plasmon-polariton modes [17]. In the THz spectral range it presents a great alternative to simple metal as it can be used to create waveguides and SPR sensors using industrial microfabrication process [18,19]. Moreover, its free carrier concentration (and, hence, surface wave optical properties) can be adjustment via temperature tuning and doping. Additionally, magnetic fields can be utilized to modulate electrical and optical proprieties of this magneto-optical material [20], thus enabling new sensor modalities.

Another example is an *n*-type and a *p*-type GaAs films produced by using two different doping types as reported [21] (Drude parameters are summarized in the Table 1).

**Figure 8:**
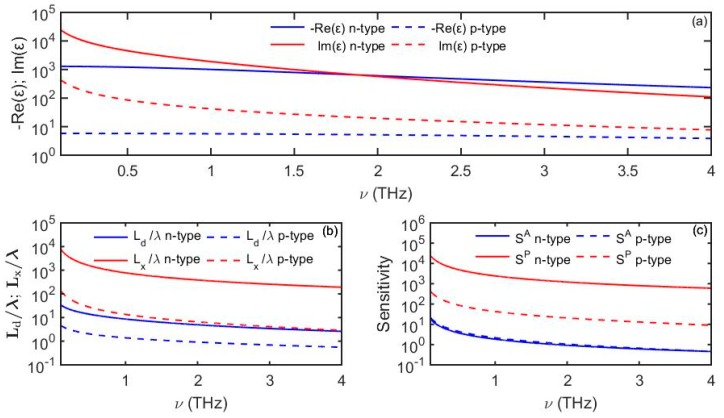
(**a**) Dielectric function of the *n*-type and *p*-type GaAs films following Drude model (Table 1). (**b**) Plasmon penetration depth into the analyte and propagation distance. (**c**) Amplitude-based sensitivity *S^A^* (1/RIU) and phase-based sensitivity *S^P^* (rad/RIU) as a function of frequency. For the analyte we use *n_d_* = 1.

In Figure 8 we present the complex dielectric function for the *n*-type and *p*-type GaAs films along with the expected plasmon propagation length and penetration depths into the analyte. We observe that the surface wave penetration depth into the analyte is smaller for the *p*-type (that has plasma frequency closer to the terahertz spectral range) than for the *n*-type. At the same time, more tightly localized surface waves propagating on the *p*-type semiconductor are lossier that those propagating on the *n*-type one, consequently, their phase sensitivity is smaller compared to that of the surface waves propagating on the *n*-type semiconductor, a behavior that is consistent with that of simple metals (see Equation (36)). We, thus, conclude that doping could be used as a design parameter to tune the surface weave probing depth into the analyte depending on the desired sensor application.

The ability to design or tune semiconductor electronic properties (such as plasma frequency and loss) make semiconductors a potent platform for surface wave sensing applications in THz spectral range.

### 5.3. Polar Materials

Now, we consider polar materials, which are characterized by the complex dielectric permittivity that also depends on the intermolecular and intramolecular resonance frequencies. The Lorentz model is typically used to model the frequency response of the dielectric function of polar materials [22]:

(45) $$\varepsilon \left(\omega \right)=\varepsilon_{\infty }\left[1-\frac{\omega_{p}^{2}}{\omega^{2}-\omega_{0}^{2}+i\gamma \omega }\right]$$

where $\varepsilon_{\infty }$ is the dielectric constant at high frequency, $\omega_{p}$ is the plasma frequency, $\omega_{0}$ is a resonance frequency and γ is the damping coefficient. The real and imaginary parts of the dielectric constant (Equation (45)) can be expressed as:

(46) $$\varepsilon^{\prime }\left(\omega \right)=\varepsilon_{\infty }\left[1-\frac{\omega_{p}^{2}\left(\omega^{2}-\omega_{0}^{2}\right)}{{\left(\omega^{2}-\omega_{0}^{2}\right)}^{2}+\gamma^{2}\omega^{2}}\right]$$

(47) $$\;\varepsilon^{\prime \prime }\left(\omega \right)=\frac{\varepsilon_{\infty }\omega_{p}^{2}\gamma \omega }{{\left(\omega^{2}-\omega_{0}^{2}\right)}^{2}+\gamma^{2}\omega^{2}}\;$$

**Figure 9:**
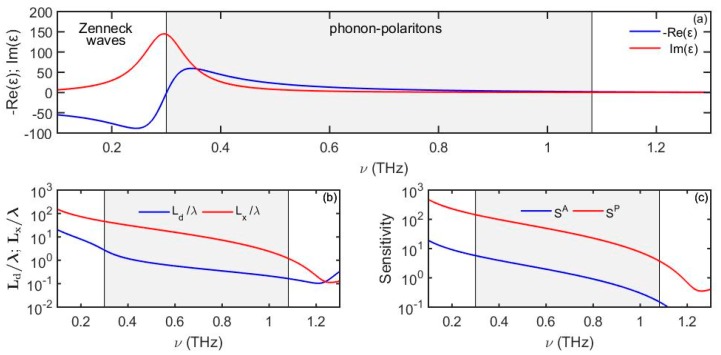
(**a**) Dielectric function of PVDF following Lorentz’s model. (**b**) Phonon-polariton penetration depth into the analyte and propagation distance. (**c**) Amplitude-based sensitivity *S^A^* (1/RIU) and phase-based sensitivity *S^P^* (rad/RIU) as a function of frequency. For the analyte we use *n_d_* = 1.

For such polar materials, the real part of the permittivity can be negative near the resonance frequency $\omega >\omega_{0}$. As the nature of the resonance, especially at THz frequencies, is often related to the vibrational movements of macromolecules or material domains, then the resultant surface waves can be called phonon polaritons. Table 2 summarizes the Lorentz parameters for some polar materials:

In Figure 9 we present dielectric properties of the PVDF material along with the phonon-polariton propagation distance and penetration depth into the analyte. We observe that the surface wave confinement is more significant at higher frequencies, especially closer to the phonon-polariton cutoff frequency at which $\varepsilon_{m}^{\prime }\left(\omega_{cutoff}\right)+\varepsilon_{d}=0$. We also note that at frequencies right below the resonance frequency $\omega_{0}$, polar material dielectric constant can have very large and positive values of both their real and imaginary parts, which is similar to the case of lossy high-k dielectrics. As a result, immediately below the resonance frequency $\omega_{0}$, polar materials support Zenneck waves, while above the resonance frequency $\omega_{0}$ they support phonon polaritons. We also observe that the surface wave probing depth into the analyte is subwavelength for the most part of the THz frequency range, thus enabling “surface” sensing with surface phonon polaritons.

### 5.4. Zero-Gap Materials

**Figure 10:**
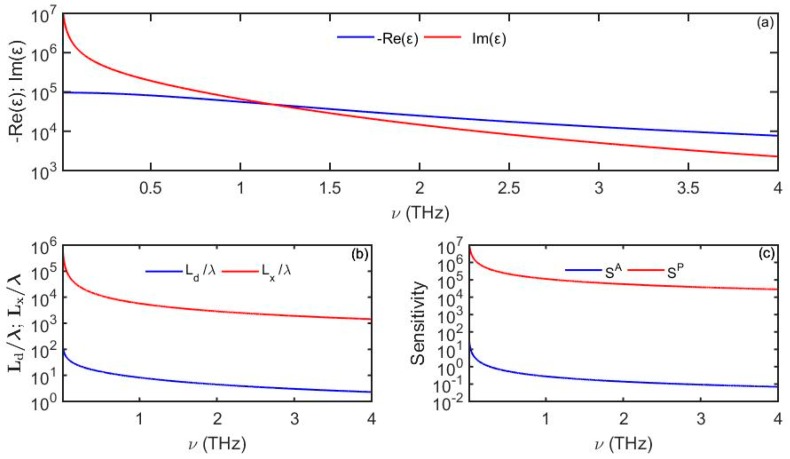
(**a**) Dielectric function of a doped monolayer of graphene. (**b**) Plasmon penetration depth into the analyte and propagation distance. (**c**) Amplitude-based sensitivity *S^A^* (1/RIU) and phase-based sensitivity *S^P^* (rad/RIU) as a function of frequency. For the analyte we use *n_d_* = 1.

Zero-gap materials are semiconductors where the conduction and valence band edges meet at the Fermi level [27]. Here, the electrons can easily change state to fill empty bands. Therefore, these materials are extremely sensitive to external influences such as pressure and magnetic field. Graphene, which consists of a 2D hexagonal arrangement of carbon atoms, is a famous example of a zero-gap semiconductor. This material, which can be tuned by a magnetic field and chemical doping, which could lead to the development of many new electronic devices [28]. The dielectric function of a monolayer of graphene is given by [29]:

(48) $$\tilde{\varepsilon }\left(\omega \right)=1-\frac{1}{\pi \hbar^{2}\varepsilon_{0}t_{g}}\frac{e^{2}\mu }{\omega \left(\omega +i\tau^{-1}\right)}$$

where $t_{g}=0.34$ nm is the thickness of the graphene monolayer, µ is the chemical potential and τ is the scattering rate. In Figure 10 we plot the dielectric properties in the THz spectral range of a doped monolayer graphene film of μ=0.135 eV and $\tau =1.35\times 10^{-13}$ s [30]. In contrast to simple metals, real part of the graphene dielectric constant is larger (in absolute value) that its imaginary part for most of the THz spectral range. Here, the plasmon-polariton confinement increases with frequency and modal size becomes comparable with the wavelength at higher frequencies (~3 THz). Due to a significant modal penetration depth into the analyte, plasmon-polariton loss is low, thus resulting in very long plasmon propagation distances.

### 5.5. Lossy High-k Dielectrics

**Figure 11:**
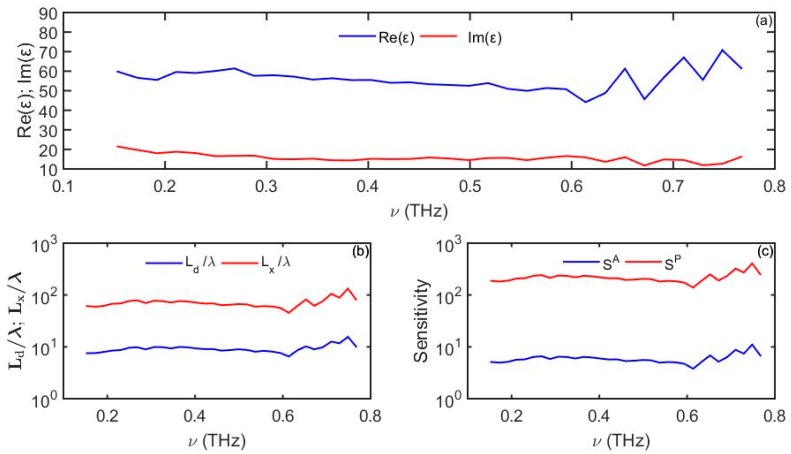
(**a**) Experimental values of the complex dielectric constant of a BST-LMT at room temperature. (**b**) Zenneck-wave penetration depth into the analyte and propagation distance. (**c**) Amplitude-based sensitivity *S^A^* (1/RIU) and phase-based sensitivity *S^P^* (rad/RIU) as a function of frequency. For the analyte we use *n_d_* = 1.

In Section 3.4 we demonstrated that a well-defined surface wave can propagate at the surface of a lossy high-k dielectric. In particular, to support a well-localized surface mode one has to require that the imaginary part of the high-k dielectric constant is comparable to its real part. As an example, in Figure 11 we present optical properties of a 0.4Ba_0.6_Sr_0.4_TiO_3_-0.6La(Mg_0.5_Ti_0.5_)O_3_ (BST-LMT) ceramic as reported [31], as well as the optical properties of a Zenneck wave that can propagate at the high-k dielectric/analyte interface. Note that in this particular example, despite the relatively large modal extent into the analyte $\left(L_{d}\sim 10\lambda \right)$, the modal propagation length is considerably longer than the modal size $\left(L_{x}\sim 70\lambda \gg L_{d}\right)$, thus a well-defined surface state is achieved at the high-k dielectric/analyte interface.

More generally, high-k dielectrics are relatively abundant in the form of various oxides and ceramics, while some of them (such as TiO_2_, Zr SnTiO_3_, MgO-TiO_2_-ZnO, Ba_2_Nd_5_Ti_9_O_27_, K_0.5_Na_0.5_NbO_3_, ZnTi_1-x_(Al_0.5_Nb_0.5_)_x_Nb_2_O_8_, etc. [32,33,34,35,36,37,38]) also feature relatively high imaginary parts of their dielectric constant compared to their real parts.

### 5.6. Artificially Structured Materials (Metamaterials)

**Figure 12:**
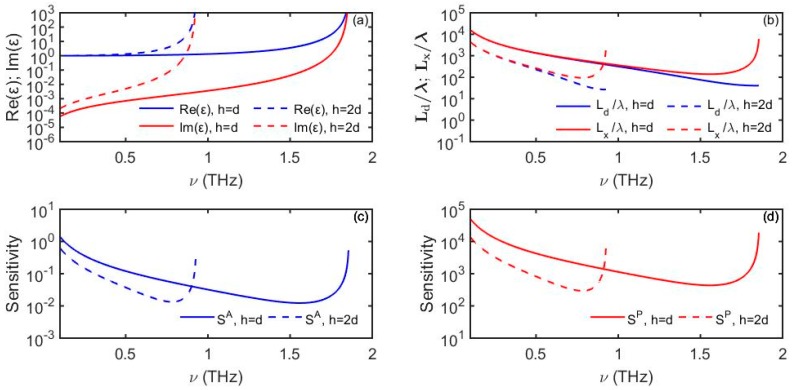
(**a**) Dielectric function of spoof surface waves supported by gold with periodic grooves (*a* = *d*/2 and *d* = 40 µm). (**b**) Spoof plasmon penetration depth into the analyte and propagation distance. (**c**) Amplitude-based sensitivity *S^A^* (1/RIU) as a function of frequency. (**d**) Phase-based sensitivity *S^P^* (rad/RIU) as a function of frequency. For analyte we use *n_d_* = 1.

Thus far, we have covered several types of naturally occurring homogeneous materials, such as simple metals, semiconductors, polar materials, zero-gap materials, and high permittivity materials that support various types of surface modes. At the same time, highly confined modes in terahertz range can also be obtained by using artificially structured materials [39]. One type of such structured material features a periodic array of subwavelength-size grooves inscribed onto a planar metallic slab in air. Thus, for a 1D array of grooves of width a, depth h, and lattice constant d (period), the complex dispersion relation of the fundamental surface mode supported by such a material (also known as a spoof surface plasmon) in the limit $a\omega n_{eff}^{spoof}/\left(2c\right)\ll 1$ can be shown to be:

(49) $$\varepsilon_{eff}^{spoof}\left(\omega \right)\approx \varepsilon_{d}+{\left(\frac{a}{d}\frac{{\tilde{\beta }}_{0}}{k_{0}}\tan\left({\tilde{\beta }}_{0}h\right)+i\frac{\varepsilon_{d}}{\sqrt{\varepsilon_{m}}}\frac{d-a}{d}\right)}^{2},$$

where:

(50) $${\tilde{\beta }}_{0}\approx k_{0}\sqrt{\varepsilon_{d}}+i\frac{\sqrt{\varepsilon_{d}}}{a\sqrt{\varepsilon_{m}}}$$

and $\varepsilon_{m}$ is the complex dielectric constant of a lossy metal. To derive the complex dispersion relation (49), one can use standard derivation together with the impedance boundary conditions at the metal surface [40]. Figure 12 shows complex dielectric function of the fundamental spoof surface plasmon $\varepsilon_{eff}^{spoof}$, its penetration depth into the analyte and propagation distance defined by Equation (19), as well as expected maximal sensitivity of an amplitude-based sensor (Equation (6)) and a phase-based sensor (Equation (11)) for two structured materials with the following geometrical parameters *h* = *d* and *h* = 2*d*, where *a* = *d*/2 and *d* = 40 µm.

At the macroscopic scale, this structured material (or metamaterial/effective medium) is analogous to a homogeneous medium that is able to support electromagnetic surface waves. Such a material can also be viewed as an artificial metal with a modified plasma frequency [41]. Many other structured materials were engineered to support surface plasmon-polariton-like modes, including 2D patterned and 3D patterned metals [42,43], helically-grooved metal wires [44], as well as dielectric-based metamaterials [45]. Moreover, more exotic waves known as Dyakonov waves can be produced at the interface with anisotropic materials which, at THz frequencies, can be, for example, in the form of periodic multilayers of two different materials featuring a deeply subwavelength period [9].

## 6. Discussion

**Figure 13:**
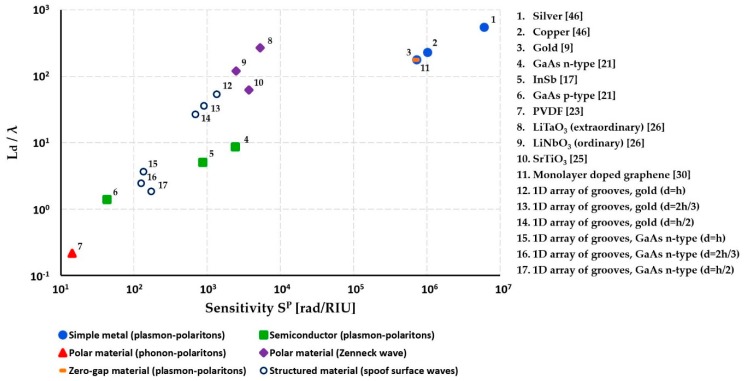
Surface wave penetration depth into the analyte against the maximal sensitivity of a phase-based sensor for various types of surface waves/materials considered in this paper at a 1 THz operation frequency. For structured materials we use *a* = *d*/2, *d* = 40 µm. For the analyte we use *n_d_* = 1 the silver and copper [46].

In the previous sections we reviewed several principal material types that allow excitation of the well-defines surface waves in the THz spectral range. Additionally, we have classified surface waves according to their physical nature and presented expressions for their penetration depth into the analyte, propagation length, as well as amplitude-based and phase-based sensitivities. For some of the surface wave types we also noted a correlation between the corresponding phase-based sensor sensitivity and probing depth into the analyte which, for plasmon polaritons on simple metals and some semiconductors, was $S^{P}\propto {\left(L_{d}/\lambda \right)}^{2}$, while for Zenneck waves on high-k lossy dielectrics. It was $S^{P}\propto L_{d}/\lambda$. For these wave types, sensors featuring smaller probing depths into the analyte (tighter modal confinement) also feature lower sensitivities. In other words, “surface” sensors (with subwavelength modal confinement) tend to have lower sensitivities than their “bulk” counterparts. In Figure 13, we summarize the article’s findings by plotting surface wave penetration depth into the analyte against the maximal sensitivity of a phase-based sensor for various types of surface waves/materials considered in this paper at a 1 THz operation frequency.

We can also define sensors with modal probing depth into the analyte above ~10 wavelengths as “bulk” sensors, while those with smaller modal sizes are defined as “surface” sensors. Thus, the main candidates as enabling materials for THz surface sensing are semiconductors with plasma frequencies in the THz range, polaritonic materials with resonance frequencies in the THz spectral range, or structured materials that support THz spoof plasmons, and they are capable of phase sensitivities in the 10–10^4^ rad/RUI range and require relatively small sensor sizes (short modal propagation length). Higher phase sensitivities 10^4^–10^7^ rad/RUI can be achieved using simple metals, zero-gap materials, semiconductors with high plasma frequency, lossy high-k dielectrics, and some structured materials, however, they operate in the bulk sensing modality with penetration depth as high as 10^2^–10^3^λ and often require large sensor sizes (long modal propagation length). Thus, depending on the sensor application modality, size limitation, and required sensitivity, a judicious choice of the surface wave type and enabling material has to be made.

It is important to note that optical properties of the surface waves are extremely sensitive to the surface preparation and presence of both material and geometrical imperfections at the interface [11,12]. Therefore, one has to be conscious that theoretical estimates presented in this paper for the wave optical properties and sensor sensitivities can be significantly different from the experimental ones. Additionally, one must be aware that different surface preparations of even the same material samples can result in significantly different optical properties of the surface waves.

We also note that in this paper we only aim at highlighting and comparing different types of “pure” surface waves that propagate along ideal planar surfaces. Clearly, optical properties of such waves can be significantly modified and augmented (lower losses, stronger confinement near the surface, etc.) when using even the simplest surface modification techniques, such as the deposition of judiciously chosen subwavelength-size dielectric layers [47,48], etc. Such techniques are important and diverse and deserve a review paper of their own.

Finally, we would like to discuss briefly an issue of excitation of the THz surface waves. Efficient excitation is generally a challenging task due to the mismatch of the propagation constants of the incident waves (which are typically launched from the side of an analyte) with that of a surface wave. Additionally, there is also an issue of spatial field matching complicated by the strong spatial inhomogeneity of the surface waves in the direction perpendicular to the interface. The simplest methods for surface wave excitation employs strongly spatially inhomogeneous fields near the interface, thus generating both propagating and evanescent fields in the near vicinity of the surface. While some of these waves can couple to the surface waves, most are lost to radiation, thus resulting in the relatively low efficiency coupling with the main advantage being simplicity of the coupling arrangement. As an example, aperture-limited plane wave has been studied in great details and used for the excitation of SPPs and Zenneck waves [47,49]. Strongly inhomogeneous fields near the interface can be obtained by using a short parallel plate waveguide parallel to the surface [48] or even a simple end-fire (butt coupling) arrangement where an incident electromagnetic field is focused onto the input facet at the position of the material/analyte interface [50]. Other methods that typically result in higher excitation efficiency of surface waves rely on careful matching of the wavevectors of an incident wave (normally from the side of an analyte) with that of a surface wave, while also employing some type of a directional coupler arrangement. A classic technique based on this principle employs a higher refractive index (compared to that of an analyte) prism coupler, where the incident angle of the excitation wave is chosen in such a way as to match the propagation constant of the incident wave (projection of the wavevector onto the surface plane) with that of a surface wave [51]. Another classic technique achieves matching of the excitation field and surface wave propagation constants by employing diffraction grating inscribed onto the surface [52]. A more exotic surface wave excitation scheme uses frequency difference generation of THz radiation near the metalized surface of a nonlinear material by employing two slightly mismatched in frequency IR lasers [53,54]. The generation of surface waves is obtained when both the wavevector and energy conservation conditions are respected between two IR laser waves and a THz surface wave. Finally, in addition to many of the methods mentioned above, excitation of surface waves on structured surfaces (spoof plasmons) was achieved by coupling to traditional waveguides via tapers or mode converters [55].

## 7. Conclusions

In this paper we reviewed two major types of sensor detection modalities (amplitude-based and phase-based) in the context of the surface wave enhanced sensing in the terahertz frequency band. A variety of surface wave types were considered including surface plasmon polaritons on metals, semiconductors, and zero gap materials, surface phonon polaritons on polaritonic materials, Zenneck waves on high-k dielectrics, as well as spoof surface plasmons and spoof Zenneck waves on structured interfaces. Special attention was paid to the tradeoff between surface wave localization and sensor sensitivity. Furthermore, a detailed theoretical analysis of the surface wave optical properties as well as the sensitivity of sensors based on such waves was supplemented with many examples related to naturally occurring and artificial materials.

## Figures and Tables

**Table 1:** Drude parameters for the n-type and p-type GaAs films.

GaAs	m * (m_0_)	*N* (cm^3^)	τ (s)	$\;\omega_{p}/2\pi \;(\mathrm{THz})$	γ/2π (THz)
*n*-type	0.079	4.48 × 10^18^	8.47 × 10^−14^	67.62	1.879
*p*-type	0.34	1.15 × 10^18^	2.51 × 10^−14^	16.51	6.341

**Table 2:** Lorentz parameters for various polar materials.

Material	$\;\varepsilon_{\infty }\;$	$\;\omega_{0}/2\pi \;(\mathrm{THz})$	$\;\omega_{p}/2\pi \;(\mathrm{THz})$	γ/2π (THz)
PVDF [23]	2.0	0.3	1.47	0.1
D-Glucose [24]	2.940	1.446	0.037	0.021
SrTiO_3_ [25]	8.528	2.632	15.77	0.6
LiNbO_3_ (ordinary) [26]	13.2	4.53	6.99	0.51
LiNbO_3_ (extraordinary) [26]	20.6	3.87	2.04	0.69
LiTaO_3_ (ordinary) [26]	13.4	4.23	6.12	0.15
LiTaO_3_ (extraordinary) [26]	8.4	5.96	11.63	0.34

## References

[1] Barnes W.L., Dereux A., Ebbesen T.W. (2003). Surface plasmon subwavelength optics. Nature.

[2] Homola J. (1995). Optical fiber sensor based on surface plasmon excitation. J. Sens. Actuators B Chem..

[3] Homola J., Yee S.S., Gauglitz G. (1999). Surface plasmon resonance sensors: Review. Sens. Actuators B Chem..

[4] Haffner C., Heni W., Fedoryshyn Y., Niegemann J., Melikyan A., Elder D.L., Bäuerle B., Salamin Y., Josten A., Koch U. (2015). All-plasmonic Mach–Zehnder modulator enabling optical high-speed communication at the microscale. Nat. Photonics.

[5] Wu L., Chu H.S., Koh W.S., Li E.P. (2010). Highly sensitive graphene biosensors based on surface plasmon resonance. Opt. Express.

[6] Guerboukha H., Nallappan K., Skorobogatiy M. (2018). Toward real-time terahertz imaging. Adv. Opt. Photonics.

[7] Abbas A., Linman M.J., Cheng Q. (2011). New trends in instrumental design for surface plasmon resonance-based biosensors. Biosens. Bioelectron..

[8] Menikh A., MacColl R., Mannella C.A., Zhang X.C. (2002). Terahertz biosensing technology: Frontiers and progress. Chemphyschem.

[9] Skorobogatiy M. (2012). Nanostructured and Subwavelength Waveguides: Fundamentals and Applications.

[10] Frezza F., Tedeschi N. (2015). Electromagnetic inhomogeneous waves at planar boundaries: Tutorial. JOSA A.

[11] Mitsushio M., Miyashita K., Higo M. (2006). Sensor properties and surface characterization of the metal-deposited SPR optical fiber sensors with Au, Ag, Cu, and Al. Sens. Actuators A Phys..

[12] Gerasimov V.V., Knyazev B.A., Lemzyakov A.G., Nikitin A.K., Zhizhin G.N. (2016). Growth of terahertz surface plasmon propagation length due to thin-layer dielectric coating. JOSA B.

[13] Bell R.J., Alexander R., Ward C., Tyler I. (1975). Introductory theory for surface electromagnetic wave spectroscopy. Surf. Sci..

[14] Mirlin D.N., Lagois J., Fischer B., Zhizhin G.N., Moskalova M.A., Shomina E.V., Yakovlev V.A., Vinogradov E.A., Yudson V.I., Agranovich V.M., Agranovich V.M., Mills D.L. (1982). Surface Polaritons: Electromagnetic Waves at Surfaces and Interfaces.

[15] Schlesinger Z., Sievers A. (1982). IR surface-plasmon attenuation coefficients for Ge-coated Ag and Au metals. Phys. Rev. B.

[16] Welford K. (1991). Surface plasmon-polaritons and their uses. Opt. Quantum Electron..

[17] Isaac T.H., Barnes W.L., Hendry E. (2008). Determining the terahertz optical properties of subwavelength films using semiconductor surface plasmons. Appl. Phys. Lett..

[18] Ma Y., Zhou J., Pištora J., Eldlio M., Nguyen-Huu N., Maeda H., Wu Q., Cada M. (2016). Subwavelength InSb-based Slot wavguides for THz transport: Concept and practical implementations. Sci. Rep..

[19] Shibayama J., Shimizu K., Yamauchi J., Nakano H. (2016). Surface plasmon resonance waveguide sensor in the terahertz regime. J. Lightwave Technol..

[20] Chochol J., Postava K., Čada M., Vanwolleghem M., Halagačka L., Lampin J.-F., Pištora J. (2016). Magneto-optical properties of InSb for terahertz applications. AIP Adv..

[21] Matsumoto N., Hosokura T., Nagashima T., Hangyo M. (2011). Measurement of the dielectric constant of thin films by terahertz time-domain spectroscopic ellipsometry. Opt. Lett..

[22] Zhang X.-C., Xu J. (2010). Introduction to THz Wave Photonics.

[23] Hidaka T., Minamide H., Ito H., Nishizawa J.-i., Tamura K., Ichikawa S. (2005). Ferroelectric PVDF cladding terahertz waveguide. J. Lightwave Technol..

[24] Sun P., Zou Y. (2016). Complex dielectric properties of anhydrous polycrystalline glucose in the terahertz region. Opt. Quantum Electron..

[25] Matsumoto N., Fujii T., Kageyama K., Takagi H., Nagashima T., Hangyo M. (2009). Measurement of the soft-mode dispersion in SrTiO3 by terahertz time-domain spectroscopic ellipsometry. Jpn. J. Appl. Phys..

[26] Schall M., Helm H., Keiding S. (1999). Far infrared properties of electro-optic crystals measured by THz time-domain spectroscopy. Int. J. Infrared Millim. Waves.

[27] Wang X.-L., Dou S.X., Zhang C. (2010). Zero-gap materials for future spintronics, electronics and optics. NPG Asia Mater..

[28] Hanson G.W. (2008). Quasi-transverse electromagnetic modes supported by a graphene parallel-plate waveguide. J. Appl. Phys..

[29] Srivastava T., Purkayastha A., Jha R. (2016). Graphene based surface plasmon resonance gas sensor for terahertz. Opt. Quantum Electron..

[30] Jablan M., Buljan H., Soljačić M. (2009). Plasmonics in graphene at infrared frequencies. Phys. Rev. B.

[31] Wu L., Jiang L., Xu Y., Ding X., Yao J. (2013). Optical tuning of dielectric properties of Ba0. 6Sr0. 4TiO3-La (Mg0. 5Ti0. 5) O3 ceramics in the terahertz range. Appl. Phys. Lett..

[32] Yu C., Zeng Y., Yang B., Donnan R., Huang J., Xiong Z., Mahajan A., Shi B., Ye H., Binions R. (2017). Titanium dioxide engineered for near-dispersionless high Terahertz permittivity and ultra-low-loss. Sci. Rep..

[33] Bolivar P.H., Brucherseifer M., Rivas J.G., Gonzalo R., Ederra I., Reynolds A.L., Holker M., de Maagt P. (2003). Measurement of the dielectric constant and loss tangent of high dielectric-constant materials at terahertz frequencies. IEEE Trans. Microw. Theory Tech..

[34] Wang S., Gu J., Han J., Zhang W. Terahertz dielectric properties of MgO-TiO 2-ZnO based ceramics. Proceedings of the 40th International Conference on Infrared, Millimeter, and Terahertz waves (IRMMW-THz).

[35] Huang J., Yang B., Yu C., Zhang G., Xue H., Xiong Z., Viola G., Donnan R., Yan H., Reece M.J. (2015). Microwave and terahertz dielectric properties of MgTiO3–CaTiO3 ceramics. Mater. Lett..

[36] Kužel P., Petzelt J. (2000). Time-resolved terahertz transmission spectroscopy of dielectrics. Ferroelectrics.

[37] Buixaderas E., Bovtun V., Kempa M., Savinov M., Nuzhnyy D., Kadlec F., Vaněk P., Petzelt J., Eriksson M., Shen Z. (2010). Broadband dielectric response and grain-size effect in K 0.5 Na 0.5 NbO 3 ceramics. J. Appl. Phys..

[38] Luo W., Li L., Yu S., Li J., Zhang B., Qiao J., Chen S. (2019). Bond theory, terahertz spectra, and dielectric studies in donor-acceptor (Nb-Al) substituted ZnTiNb2O8 system. J. Am. Ceram. Soc..

[39] Shen X., Cui T.J., Martin-Cano D., Garcia-Vidal F.J. (2013). Conformal surface plasmons propagating on ultrathin and flexible films. Proc. Natl. Acad. Sci. USA.

[40] Rusina A., Durach M., Stockman M.I. (2010). Theory of spoof plasmons in real metals. Appl. Phys. A.

[41] Ng B., Wu J., Hanham S.M., Fernández-Domínguez A.I., Klein N., Liew Y.F., Breese M.B., Hong M., Maier S.A. (2013). Spoof plasmon surfaces: A novel platform for THz sensing. Adv. Opt. Mater..

[42] Zhu W., Agrawal A., Nahata A. (2008). Planar plasmonic terahertz guided-wave devices. Opt. Express.

[43] Pors A., Moreno E., Martin-Moreno L., Pendry J.B., Garcia-Vidal F.J. (2012). Localized spoof plasmons arise while texturing closed surfaces. Phys. Rev. Lett..

[44] Fernández-Domínguez A., Williams C.R., García-Vidal F., Martín-Moreno L., Andrews S.R., Maier S. (2008). Terahertz surface plasmon polaritons on a helically grooved wire. Appl. Phys. Lett..

[45] Zhao Q., Zhou J., Zhang F., Lippens D. (2009). Mie resonance-based dielectric metamaterials. Mater. Today.

[46] Johnson P.B., Christy R.-W. (1972). Optical constants of the noble metals. Phys. Rev. B.

[47] Gong M., Jeon T.-I., Grischkowsky D. (2009). THz surface wave collapse on coated metal surfaces. Opt. Express.

[48] Gerasimov V.V., Knyazev B.A., Kotelnikov I.A., Nikitin A.K., Cherkassky V.S., Kulipanov G.N., Zhizhin G.N. (2013). Surface plasmon polaritons launched using a terahertz free-electron laser: Propagation along a gold–ZnS–air interface and decoupling to free waves at the surface edge. JOSA B.

[49] Ling R., Scholler J., Ufimtsev P.Y. (1998). The propagation and excitation of surface waves in an absorbing layer. Prog. Electromagn. Res..

[50] Stegeman G., Wallis R., Maradudin A. (1983). Excitation of surface polaritons by end-fire coupling. Opt. Lett..

[51] Nazarov M.M., Shkurinov A.P., Garet F., Coutaz J.-L. (2015). Characterization of highly doped Si through the excitation of THz surface plasmons. IEEE Trans. Terahertz Sci. Technol..

[52] Li S., Jadidi M.M., Murphy T.E., Kumar G. (2013). Terahertz surface plasmon polaritons on a semiconductor surface structured with periodic V-grooves. Opt. Express.

[53] Kumar P., Tripathi V. (2013). Terahertz surface plasmon excitation via nonlinear mixing of lasers in a metal-coated optical fiber. Opt. Lett..

[54] Singh R., Tripathi V. (2016). Laser excitation of terahertz surface plasma wave over a hollow capillary plasma. Laser Part. Beams.

[55] Liu L., Li Z., Gu C., Xu B., Ning P., Chen C., Yan J., Niu Z., Zhao Y. (2015). Smooth bridge between guided waves and spoof surface plasmon polaritons. Opt. Lett..

